# Pioglitazone leads to an inactivation and disassembly of complex I of the mitochondrial respiratory chain

**DOI:** 10.1186/1741-7007-11-88

**Published:** 2013-08-01

**Authors:** Inmaculada García-Ruiz, Pablo Solís-Muñoz, Daniel Fernández-Moreira, Teresa Muñoz-Yagüe, José A Solís-Herruzo

**Affiliations:** 1 Research Center, Laboratory of Gastroenterology and Hepatology, University Hospital ‘12 de Octubre’, Complutense University, Madrid 28041, Spain; 2 Centro de Investigación Hospital Universitario ‘12 de Octubre’, Avenida de Córdoba S/N, Madrid, 28041, Spain; 3 Institute of Liver Studies, King’s College Hospital, London SE5 9RS, UK; 4 Department of Bromatology and Food Hygiene, Military Center of Veterinary of Defense, Madrid 28024, Spain

**Keywords:** ATP, Mitochondrial respiratory chain, Pioglitazone, Proteomic, Thiazolidinediones

## Abstract

**Background:**

Thiazolidinediones are antidiabetic agents that increase insulin sensitivity but reduce glucose oxidation, state 3 respiration, and activity of complex I of the mitochondrial respiratory chain (MRC). The mechanisms of the latter effects are unclear. The aim of this study was to determine the mechanisms by which pioglitazone (PGZ), a member of the thiazolidinedione class of antidiabetic agents, decreases the activity of the MRC. In isolated mitochondria from mouse liver, we measured the effects of PGZ treatment on MRC complex activities, fully-assembled complex I and its subunits, gene expression of complex I and III subunits, and [^3^H]PGZ binding to mitochondrial complexes.

**Results:**

*In vitro,* PGZ decreased activity of complexes I and III of the MRC, but *in vivo* only complex I activity was decreased in mice treated for 12 weeks with 10 mg/kg/day of PGZ. *In vitro* treatment of isolated liver mitochondria with PGZ disassembled complex I, resulting in the formation of several subcomplexes. In mice treated with PGZ, fully assembled complex I was increased and two additional subcomplexes were found. Formation of supercomplexes CI+CIII_2_+CIV_n_ and CI+CIII_2_ decreased in mouse liver mitochondria exposed to PGZ, while formation of these supercomplexes was increased in mice treated with PGZ. Two-dimensional analysis of complex I using blue native/sodium dodecyl sulfate polyacrylamide gel electrophoresis (BN/SDS-PAGE) showed that *in vitro* PGZ induced the formation of four subcomplexes of 600 (B), 400 (C), 350 (D), and 250 (E) kDa, respectively. Subcomplexes B and C had NADH:dehydrogenase activity, while subcomplexes C and D contained subunits of complex I membrane arm. Autoradiography and coimmunoprecipitation assays showed [^3^H]PGZ binding to subunits NDUFA9, NDUFB6, and NDUFA6. Treatment with PGZ increased mitochondrial gene transcription in mice liver and HepG2 cells. In these cells, PGZ decreased intracellular ATP content and enhanced gene expression of *specific protein 1* and *peroxisome-proliferator activated receptor* (*PPAR*)*γ coactivator 1α* (*PGC-1α*).

**Conclusions:**

PGZ binds complex I subunits, which induces disassembly of this complex, reduces its activity, depletes cellular ATP, and, in mice and HepG2 cells, upregulates nuclear DNA-encoded gene expression of complex I and III subunits.

## Background

Pioglitazone (PGZ), a member of the thiazolidinedione (TZD) class of antidiabetic agents and agonist of the peroxisome proliferator-activated receptor γ (PPARγ) [1], improves insulin sensitivity both in the liver and peripheral tissues. The mechanisms of this effect are unclear. However, they are attributed to the actions of TZDs on PPARγ [2]. After activating PPARγ receptors, TZDs induce adipocyte differentiation and remodeling of adipose tissue *in vitro* and *in vivo*[2,3]. It is believed that signals derived from the adipose tissue (fatty acids, adiponectin, resistin, leptin) may mediate the improvement in skeletal glucose disposal induced by TZDs [2,3]. However, it is also likely that other mechanisms independent of PPARγ may contribute to TZD effects on insulin sensitivity [4]. Thus, *in vitro* studies have shown that TZDs elevate lactate production by skeletal muscle [5], suggesting an inhibition of cell respiration [6]. In fact, several authors have found that the activity of complex I of the mitochondrial respiratory chain (MRC), state 3 respiration, and glucose oxidation were reduced in homogenates of skeletal muscle treated with increased doses of TZDs [7,8]. PPARγ does not seem to be involved in these effects of TZDs [5], since they inhibited complex I in sonicated tissue homogenates containing disrupted mitochondria [7].

Complex I (NADH:ubiquinone oxidoreductase) is the first and the largest of the four multiprotein complexes that constitute the MRC involved in oxidative phosphorylation [9]. This complex is formed by at least 44 subunits, 7 of which are encoded by the mitochondrial genome and the remaining 37 by the nuclear genome [10]. The crystal structure of the entire *Thermus thermophilus* complex I has been recently reported [11]. In previous studies, we have shown that PGZ suppressed the activity of complex I of the MRC in ob/ob mice, but the mechanisms of this effect are still unclear [12].

The aim of the present study was to determine the mechanisms by which PGZ decreases MRC activity. We show that PGZ binds subunits located in the membrane arm of complex I of the MRC, which induces disassembly of this complex, reduces its enzymatic activity, depletes cellular ATP, and consequently upregulates nuclear DNA-encoded gene expression of complex I subunits.

## Results

### PGZ decreased activity of complexes I and III of the MRC in isolated mouse liver mitochondria

**Figure 1:**
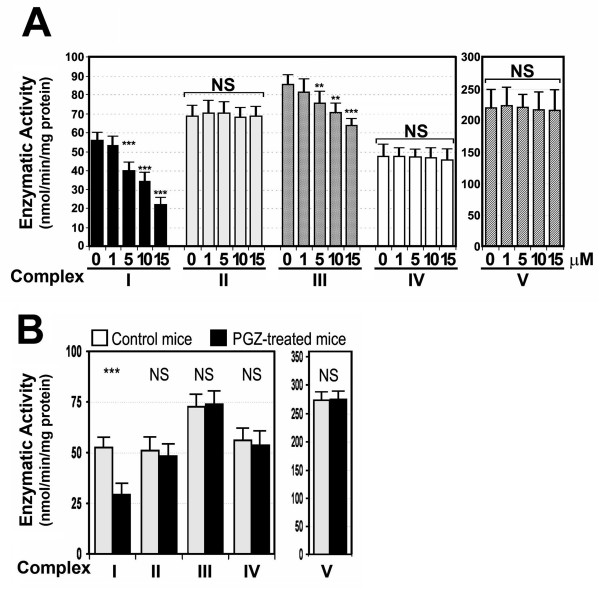
**Pioglitazone (PGZ) decreased enzymatic activity of complex I and III of the mitochondrial respiratory chain. (A)** The *in vitro* enzymatic activity of mitochondrial respiratory chain (MRC) complexes was measured by spectrophotometry (as described in the Methods section) in isolated mouse liver mitochondria exposed to 0 to 15 μM PGZ for 30 minutes. Activities were expressed as nM of substrate used per minute per mg protein and referred to as the percentage of the specific activity of citrate synthase (CS) to correct for the hepatic content in mitochondria. NS, not significant. **, *P* <0.001; ***, *P* <0.001 vs control activity. **(B)** Effects of PGZ on the enzymatic activity of the MRC and complex V of the oxidative phosphorylation in mice treated with PGZ for 12 weeks. Ten C57BL/6J mice were treated with 10 mg/kg/day PGZ for 12 weeks. The enzymatic activity of MRC complexes was measured as indicated in the Methods section and expressed as nM of substrate used per minute per mg protein and, to correct for the hepatic content in mitochondria, referred as a percentage of the specific activity of citrate synthase (CS). NS, not significant.

As the MRC plays a critical role in the conversion of NADH and FADH2 into NAD and FAD, respectively, and in the generation of ATP from ADP [13], we measured the *in vitro* effect of increasing concentrations (0 to 15 μM) of PGZ on the activity of MRC complexes isolated from mouse liver. The activity of complex I, which accepts electrons from NADH and transfers them to ubiquinone, decreased in a dose-dependent manner from 55.67 ± 3.7 nmol/min/mg protein in untreated mitochondria (100%) to 21.98 ± 4.3 nmol/min/mg protein (39.5 ± 0.5%) in mitochondria treated with 15 μM PGZ for 30 minutes (Figure 1A). To correct for mitochondrial volume, all respiratory chain enzyme activities were normalized to the activity of citrate synthase (CS).

The activity of complex II (succinate dehydrogenase complex), which passes electrons directly to ubiquinone, was not affected significantly by PGZ treatment (control, 68.96 ± 4.8 nmol/min/mg protein; 15 μM PGZ, 67.96 ± 5.3 nmol/min/mg protein) (Figure 1A).

Ubiquinone passes electrons from complex I and II to the b-c1 complex (complex III), which transfers them to cytochrome c. The activity of complex III also significantly decreased from 85.71 ± 4.6 nmol/min/mg protein (control) to 64.20 ± 3.7 nmol/min/mg protein (*P* <0.001) (15 μM PGZ).

Cytochrome c is involved in carrying electrons from the b-c1 complex to the cytochrome oxidase complex (complex IV), which finally transfers these electrons to oxygen. Measurement of the activity of this complex in liver mitochondria exposed to increasing concentrations of PGZ revealed that this drug did not affect complex IV activity (controls, 47.21 ± 6.1 nmol/min/mg protein; 15 μM PGZ, 45.61 ± 5.7 nmol/min/mg protein; not significant (NS)) (Figure 1A).

ATP synthase or complex V of the oxidative phosphorylation pathway converts ADP into ATP when protons flow back from the intermembrane space into the mitochondrial matrix. The activity of this complex was not significantly affected by treatment with PGZ (controls, 223 ± 38.6 nmol/min/mg protein; 15 μM PGZ, 215.7 ± 33.1 nmol/min/mg protein; NS) (Figure 1A).

### Treatment of mice with PGZ decreased activity of complex I of the MRC

We also studied the effects of PGZ on the activity of MRC complexes in C57BL/6J mice. In these animals, treatment with 10 mg PGZ/kg/day for 12 weeks significantly decreased the activity of complex I (Figure 1B) from 52.28 ± 7.16 nmol/min/mg protein in control animals to 29.32 ± 4.62 nmol/min/mg protein in PGZ-treated mice (*P* <0.001), but not the activity of complexes II (control, 50.81 ± 6.74 nmol/min/mg protein; PGZ, 48.23 ± 5.33 nmol/min/mg protein; NS), III (control, 72.70 ± 8.3 nmol/min/mg protein; PGZ, 73.92 ± 6.28 nmol/min/mg protein; NS), IV (control, 56.13 ± 6.81 nmol/min/mg protein; PGZ, 53.53 ± 7.11 nmol/min/mg protein; NS), and V (274.46 ± 36.27 nmol/min/mg protein; PGZ, 275.17 ± 31.63 nmol/min/mg protein; NS) (Figure 1B).

### Treatment with PGZ disassembled complex I *in vitro* and *in vivo*

**Figure 2:**
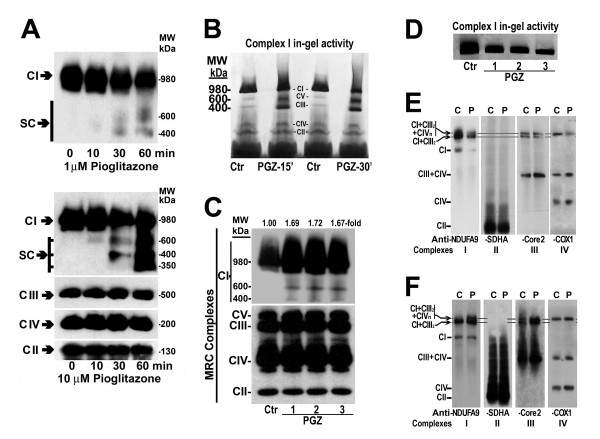
**Pioglitazone (PGZ) disassembled complex I of the mitochondrial respiratory chain and altered supercomplex formation. (A)** Mitochondria isolated from a mouse liver were treated with PGZ and analyzed as described in the Methods section. Proteins were separated using blue native polyacrylamide gel electrophoresis (BN-PAGE). Western blot analysis was performed using antibody against complex I (CI) subunit NDUFA9, complex II (CII) subunit SDHA, complex III (CIII) subunit core 2, and complex IV (CIV) subunit COX4. SC, subcomplexes. **(B)** Mouse mitochondria were incubated with PGZ and proteins were separated by BN-PAGE. Complex I ingel activity was displayed as described in the Methods section. Lanes: Ctr, control activity; PGZ, isolated mitochondria treated with 10 μM PGZ for 15 or 30 minutes. **(C)** Mitochondrial complexes isolated from an untreated mouse and three mice treated with 10 mg/kg/day PGZ for 12 weeks were run under native conditions on a BN-PAGE system and analyzed by western blot. Lanes: Ctr, control mouse; lane PGZ-1, PGZ-2, PGZ-3, PGZ-treated mice. **(D)** Mitochondrial complexes isolated as indicated in **(C)** were subjected to a complex I in-gel enzyme activity assay as described in Figure 2B. **(E)** Mouse liver mitochondria were solubilized in 4 g/g protein digitonin and complexes and supercomplexes were separated by BN-PAGE. Complexes and supercomplexes were identified by western blot using specific antibodies against subunits NDUFA9, SDHA, core 2, and COX1. CI+CIII_2_+CIVn, supercomplex formed by complex I, complex III dimer, and complex IV. CI+CIII_2_, supercomplex formed by complex I and complex III dimer. C, untreated mitochondria; P, PGZ-treated mitochondria. **(F)** Liver mitochondria isolated from PGZ-treated (P) and untreated **(C)** mice were solubilized in digitonin and complexes and supercomplexes were separated on a BN-PAGE gel. Complexes and supercomplexes were identified by western blot using antibodies described in **(E)**. These results are representative of three controls and three PGZ-treated mice.

In order to know whether PGZ alters assembly of MRC complexes, we exposed 30 μg of isolated mitochondria from normal liver to either 1 or 10 μM PGZ for 0 to 60 minutes. As Figure 2A shows, PGZ decreased the band corresponding to the fully assembled complex I and determined the formation of several lower molecular weight bands (600, 400, 350 kDa) in which the NDUFA9 subunit was also present. These effects were dose and time dependent. These changes were more evident at 60 minutes of treatment with 10 μM PGZ. As Figure 2B shows, two of these subcomplexes (600 and 400 kDa) maintained in-gel enzymatic activity. Treatment of liver mitochondria with 10 μM PGZ did not affect assembly of complexes II, III, and IV (Figure 2A).

To determine whether PGZ treatment also alters assembly of MRC complexes in mice, liver mitochondria isolated from C57BL/6J mice untreated and treated for 12 weeks with 10 mg/kg/day PGZ via intragastric intubation were separated using a one-dimensional blue native polyacrylamide gel electrophoresis (BN-PAGE) system and analyzed by western blot using antibody against the complex I subunit NDUFA9, complex II subunit SDHA, complex III subunit core 2 (UQCRC2), complex IV subunit COX4, and complex V subunit ATP5A1. As Figure 2C illustrates, animals treated with PGZ displayed a clear increase in the fully-assembled complex I as compared to untreated, control mice. In addition, similar to *in vitro* studies, two other subcomplexes of 600 and 400 kDa, respectively, were detected. The bands corresponding to the remaining four complexes were not affected by PGZ treatment. Although fully assembled complex I was increased in PGZ-treated mice, its in-gel activity was clearly decreased (Figure 2D).

### PGZ altered formation of respiratory supercomplexes

To investigate how PGZ affects the formation of respiratory supercomplexes, we performed BN-PAGE using the detergent digitonin instead of *n*-dodecylmaltoside. This condition allows loose interaction to remain intact. We found that complex I was present in the monomeric form and in two additional supercomplexes also containing complex III dimer (CI+CIII_2_) or complex III dimer and complex IV (CI+CIII_2_+CIV_n_). Treatment of liver mitochondria with 10 μM PGZ led to a marked decrease in the monomeric complex I and in both supercomplexes CI+CIII_2_ and CI+CIII_2_+CIV_n_. This decrease in supercomplex formation was due to a reduced accumulation of complexes I, III, and IV (Figure 2E). Moreover, formation of supercomplex CIII+CIV was increased after PGZ treatment.

Compared to untreated mice, in liver mitochondria isolated from mice treated with 10 mg/kg/day PGZ for 12 weeks, we found that monomeric complex I was unchanged and supercomplexes CI+CIII_2_+CIV_n_ and CI+CIII_2_ were markedly increased. Likewise, supercomplex CIII+CIV was also augmented (Figure 2F).

### PGZ disassembled the membrane arm of complex I

**Figure 3:**
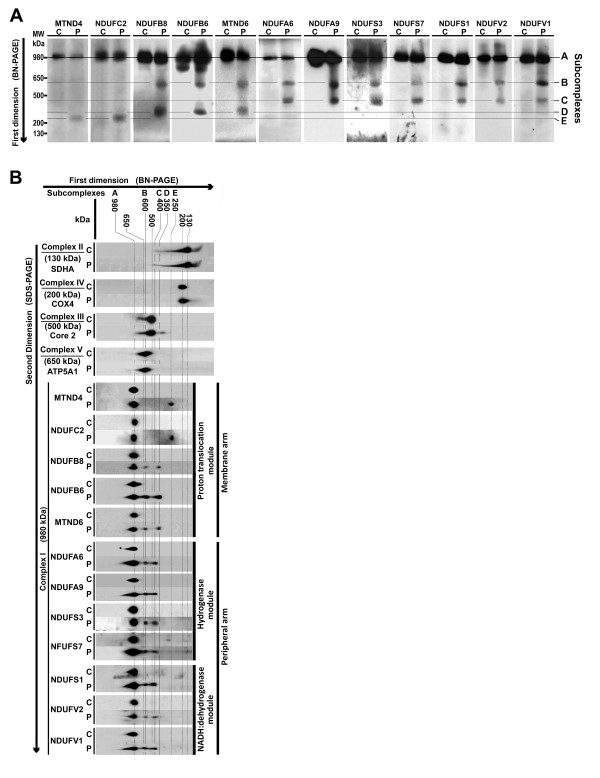
**Pioglitazone (PGZ) disassembled complex I into four subcomplexes of approximately 600, 400, 350, and 250 kDa, respectively. (A)** Mitochondrial complexes were treated with 10 μM PGZ as described in Methods and Figure 2A, separated on one-dimensional 5% to 15% blue native polyacrylamide gel electrophoresis (BN-PAGE), and analyzed by western blot using antibodies against subunits NDUFA9, NDUFA6, NDUFV1, NDUFV2, NDUFS1, NDUFS7, NDUFS3, NDUFB6, NDUFB8, NDUFC2, MTND4, and MTND6. Molecular weight was determined by the mobility of fully assembled complex I (980 kDa), complex V (650 kDa), complex III (500 kDa), complex IV (200 kDa), and complex II (130 kDa). **(B)** Mouse liver mitochondria (30 μg) were exposed to 10 μM PGZ for 30 minutes and separated in the first dimension using a 5% to 15% BN-PAGE gel and in the second dimension using sodium dodecyl sulfate (SDS)-PAGE. Subunits were identified by western blot using specific antibodies against subunits NDUFA9, NDUFA6, NDUFV1, NDUFV2, NDUFS1, NDUFS7, NDUFS3, NDUFB6, NDUFB8, NDUFC2, MTND6, and MTND4 of complex I, as well as antibodies against subunits SDHA of complex II, core 2 of complex III, COX4 of complex IV, and subunit ATP5A1 of complex V. These results are representative of three separate experiments.

With the intention of characterize subcomplexes formed after *in vitro* treatment of isolated mitochondria with 10 μM PGZ, we analyzed the presence of representative subunits of intermediates in the assembly process following the model proposed by Mimaki *et al*. [9]. Thus, after PGZ treatment, MRC complexes were separated as previously described using first-dimension BN-PAGE, blotted, and analyzed by western blot using antibodies against subunits of complex I NDUFA9, NDUFA6, NDUFV1, NDUFV2, NDUFS1, NDUFS3, NDUFS7, NDUFB6, NDUFB8, NDUFC2, MTND4, and MTND6. As Figure 3A shows, in addition to the 980 kDa fully assembled complex I, we found subunits NDUFV1, NDUFV2, NDUFS1, NDUFA9, NDUFS3, NDUFS7, MTND6, NDUFB6, NDUFB8, and NDUFA6 in an approximately 600 kDa molecular weight subcomplex B. Likewise, subunits NDUFV1, NDUFV2, NDUFS1, NDUFA9, NDUFS3, NDUFS7, and NDUFA6, but not MTND6, NDUFB6, and NDUFB8, were found in a 400 kDa subcomplex C. In an approximately 350 kDa subcomplex D, subunits MTND6, NDUFB6, and NDUFB8 were present, all of them belonging to the membrane arm of complex I. Finally, subunits NDUFC2 and MTND4 were found in an approximately 250 kDa subcomplex E.

Two-dimensional analysis of complex I confirmed that treatment of liver mitochondria with 10 μM PGZ for 30 minutes disassembled complex I as indicated by the presence of its subunits in the mature fully assembled complex (Figure 3B, subcomplex A) and four additional subcomplexes of about 600, 400, 350, and 250 kDa, respectively (Figure 3B, subcomplexes B, C, D, and E). Subcomplex B contained subunits NDUFV1, NDUFV2, and NDUFS1 that are part of the NADH:dehydrogenase module, NDUFB6, NDUFB8, and MTND6, belonging to the membrane arm of the complex, and subunits NDUFA9, NDUFA6, NDUFS3, and NDUFS7 forming part of the hydrogenase module of complex I. Subunits NDUFV1, NDUFV2, and NDUFS1 and subunits NDUFA9, NDUFA6, NDUFS3, and NDNFS7 belonging to the hydrogenase module were present in subcomplex C. The subcomplex D of about 350 kDa contained at least subunits MTND6, NDUFB6, and NDUFB8, all of them forming part of the membrane arm. Finally, subcomplex E contained at least subunits NDUFC2 and MTND4, also members of the membrane arm.

Examination of representative subunits of complexes II (SDHA), III (UQCRC2 = core 2), IV (COX4), and V (ATP5A1) showed that only subunit core 2 was modified by treatment with PGZ (Figure 3B). Thus, this subunit appeared distributed in one additional subcomplex of about 300 kDa.

### PGZ decreased prohibitin and assembly factors NDUFAF1 (CIA30), and FOXRED1 in mouse liver mitochondria

**Figure 4:**
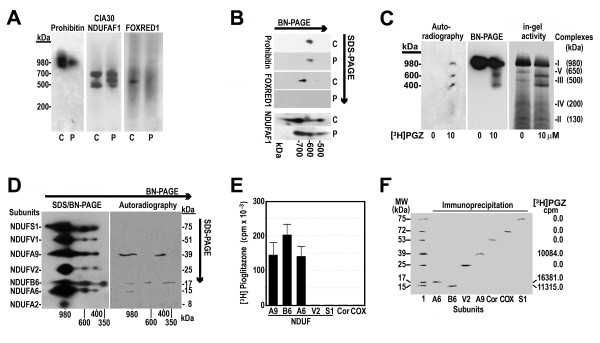
**Pioglitazone (PGZ) decreased the amount of prohibitin, NDUFAF1, and FOXRED1 associated with mitochondrial complexes and joined subunits NDUFA9, NDUFB6, and NDUFA6 of complex I.** Mouse mitochondria were treated with PGZ, proteins were separated by either blue native polyacrylamide gel electrophoresis (BN-PAGE) **(A)** or sodium dodecyl sulfate (SDS)/BN-PAGE **(B)** and analyzed by western blot using antibodies against prohibitin, NDUFAF1, or FOXRED1. C, control, untreated proteins. P, PGZ treated proteins. **(C)** Mouse liver mitochondria were treated with 10 μM [^3^H]PGZ for 30 minutes. Mitochondrial complexes were separated in parallel on three one-dimensional BN-PAGE gels. One gel was used to localize [^3^H]PGZ by autoradiography, the second gel was utilized to analyze complex I by western blot, and the third was employed to determine complex I in-gel activity. **(D)** Mitochondrial complexes were separated in parallel on two SDS/BN-PAGE gels. One gel was analyzed by western blot using antibodies against complex I subunits NDUFS1, NDUFV1, NDUFA9, NDUFV2, NDUFB6, NDUFA6, and NDUFA2. The second gel was used to localize [^3^H]PGZ by autoradiography. **(E)** Liver mitochondria treated with 10 μM [^3^H]PGZ for 30 minutes were immunoprecipitated with monoclonal antibody against NDUFA9 (A9), NDUFB6 (B6), NDUFA6 (A6), NDUFV2 (V2), NDUFS1 (S1), core 1 (Cor), and COX1 (COX) as described in the Methods section. Radioactivity was quantitated in the immunoprecipitates. **(F)** Mitochondrial proteins treated with 10 μM [^3^H]PGZ for 30 minutes were immunoprecipitated as indicated in (E), electrophoresed in a 10% acrylamide gel, and transferred to a nitrocellulose membrane. Subunits were analyzed by western blot to show specific immunoprecipitation of subunits. Individual bands were excised and their radioactivity was measured. Lane 1, western blot of not immunoprecipitated mitochondrial proteins. Lanes A6, B6, V2, A9, Cor, COX, and S1 western blot of immunoprecipitated NDUFA6, NDUFB6, NDUFV2, NDUFA9, core 1, COX1, and NDUFS1 subunits, respectively.

Because it is known that a number of proteins are involved in maintaining the stability of complex I, we investigated whether *in vitro* isolated mitochondria treatment with 10 μM PGZ for 30 minutes affected mitochondrial prohibitin, NDUFAF1 (CIA30), or FOXRED1, three proteins involved in the assembly/stability of complex I. As Figure 4 shows, the amount of prohibitin clearly decreased in mitochondrial protein exposed to PGZ (Figure 4A). Similarly, for NDUFAF1 (which appeared distributed in two separated bands of about 700 kDa and 500 kDa, respectively, in untreated proteins), we found that after PGZ treatment these two signals were less intense and a third band of about 600 kDa appeared between them (Figure 4A). Finally, FOXRED1, that originated a signal at 500 kDa of molecular weight in untreated proteins, was not recognized in PGZ-treated proteins (Figure 4A). Likewise, second-dimension analysis of NDUFAF1 and FOXRED1 proteins via sodium dodecyl sulfate (SDS)-PAGE confirmed these effects of PGZ. Thus, after PGZ treatment, the signal for FOXRED1 disappeared completely, and NDUFAF1 was distributed in three spots (Figure 4B).

### [^3^H]PGZ binds to complex I subunits NDUFA9, NDUFB6, and NDUFA6

To investigate whether PGZ interacts with complex I or with any of its subunits, we exposed isolated liver mitochondria to 10 μM [^3^H]PGZ for 30 minutes. As shown in Figure 4C, [^3^H]PGZ binds to mitochondrial proteins of about 980, 600, and 400 kDa molecular weight. To identify subunits that PGZ was bound to, we treated isolated mitochondria with 10 μM [^3^H]PGZ and complex I subunits were separated in parallel on two SDS/BN-PAGE gels as indicated in Methods. Autoradiography showed that [^3^H]PGZ was bound particularly to a subunit of 39 kDa, but also to subunits of 17 and 15 kDa that overlapped the signals originated by NDUFA9, NDUFB6, and NDUFA6, respectively (Figure 4D). The binding of [^3^H]PGZ to these subunits was confirmed by immunoprecipitating mitochondrial subunits after treating isolated mitochondria with 10 μM [^3^H]PGZ for 30 minutes (Figure 4E,F).

### Treatment with PGZ increased mitochondrial gene transcription in mice liver and HepG2 cells

**Figure 5:**
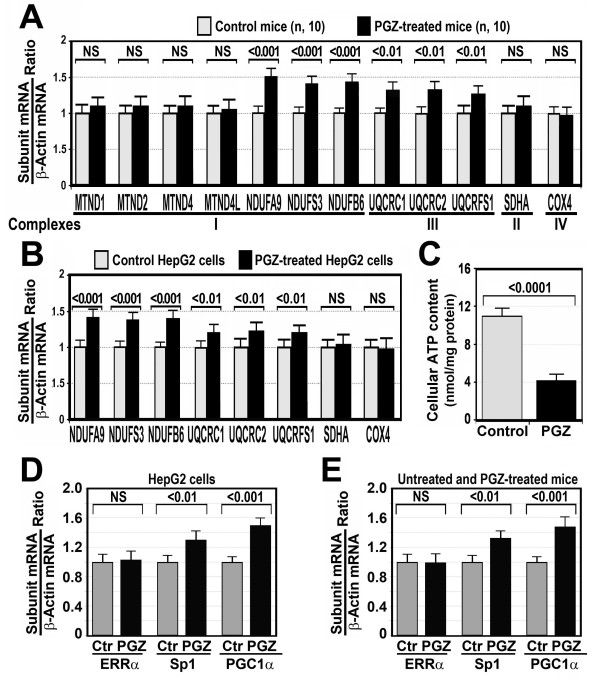
**Pioglitazone (PGZ) upregulated mitochondrial respiratory chain (MRC) subunit gene expression in C57BL/6J mice and HepG2 cells. (A)** Gene expression of representative subunits of complex I (*MTND1, MTND2, MTND4, MTND4L, NDUFA9, NDUFS3, NDUFB6*), complex II (*SDHA*), complex III (*UQCRC1*, *UQCRC2*, *UQCRF1*), complex IV (*COX4*) subunits, and *β-actin* was measured by quantitative real-time polymerase chain reaction (RT-PCR) in the liver from ten PGZ-treated and ten untreated C57BL/6J mice. The subunit mRNA/β-actin mRNA ratio was calculated. Data are expressed as fold change over control mice. NS, not significant. **(B)** Gene expression of subunits of complex I (*NDUFA9, NDUFS3, NDUFB6*), II (*SDHA*), III (*UQCRC1*, *UQCRC2, UQCRF1*), and IV (*COX4*) was measured in HepG2 cells treated with 10 μM PGZ for 5 days. Messenger RNA (mRNA) of subunits was analyzed by RT-PCR following the procedure described in the Methods section. The subunit/β-actin mRNA ratio was calculated. Data are expressed as fold change over control cells. NS, not significant. **(C)** ATP content in HepG2 cells treated with 10 μM PGZ for 5 days. Data are representative of one experiment that was repeated by quintuplicate four times with similar results. **(D**,**E)** Effects of PGZ on *estrogen-related receptor α* (*ERRα*), *specific protein 1* (*Sp1*) and *peroxisome-proliferator activated receptor* (*PPAR)γ coactivator 1α* (*PGC-1α*) gene expression in HepG2 cells and mice. (D) HepG2 cells were treated with 10 μM PGZ for 5 days. Messenger RNAs of ERRα, Sp1, and PGC1α were analyzed by RT-PCR following the procedure described in the Methods section. The subunit/β-actin mRNA ratio was calculated. Data are expressed as fold change over control cells. NS, not significant. (E) *ERRα*, *Sp1*, and *PGC1α* gene expression was analyzed by RT-PCR in the liver of mice treated with 10 mg/kg/day PGZ for 12 weeks. NS, not significant.

Because fully assembled complex I was increased in mice treated with PGZ for 12 weeks, we examined the steady-state levels of nuclear DNA (nDNA)-encoded (*NDUFA9*, *NDUFB6*, *NDUFS3, core 2*) mRNA and mitochondrial DNA (mtDNA)-encoded (*ND1*, *ND2*, *ND4, ND4L*) mRNA in the liver from ten control mice and in ten mice treated with 10 mg PGZ/kg/day for 12 weeks. This experiment revealed that gene expression of complex I subunits encoded by the nDNA was increased by 30% to 50% over control levels in PGZ-treated mice (Figure 5A), whereas expression of mtDNA encoded complex I subunits remained at the control levels in PGZ-treated mice. Gene expression of complex III subunit core 2 was also significantly increased in these mice.

To ensure that PGZ increases gene expression of MRC subunits, we treated HepG2 cells with 10 μM PGZ for 5 days and measured expression of complex I subunits *NDUFA9*, *NDUFB6*, *NDUFS3*, complex III subunits *UQCRC1*, *UQCRC2*, and *UQCRFS1*, complex IV subunit *COX4*, and complex II subunit *SDHA*. As Figure 5B shows, treatment of cells with PGZ increased gene expression of complex I subunits by 30% and complex III by 20% to 30%, but did not increase significantly gene expression of complex II and IV subunits.

### PGZ reduced intracellular ATP content and upregulated gene expression of *specific protein 1* (*Sp1*) and *PPARγ coactivator 1α* (*PGC-1α*) in HepG2 cells

To determine whether PGZ modifies intracellular ATP content, HepG2 cells were cultured in the absence or presence of 10 μM PGZ for 5 days. As Figure 5C shows, treatment of cells with 10 μM PGZ decreased cellular ATP from 10.93 ± 0.85 nmol/mg protein to 4.18 ± 0.42 nmol/mg protein (*P* <0.0001). To examine whether PGZ modifies gene expression of transcription factors involved in the regulation of gene encoding MRC proteins, we exposed HepG2 cells to 10 μM PGZ for 5 days and measured gene expression of *Sp1*, *estrogen related receptor α* (*ERRα*), and *PGC-1α* of the MRC by real-time polymerase chain reaction (RT-PCR). We found that PGZ treatment increased significantly gene expression of *Sp1* and *PGC-1α,* but not of *ERRα* (Figure 5D).

## Discussion

Our results shows that *in vitro* PGZ significantly inhibits complex I activity and, less intensely but also significantly, complex III in isolated liver mitochondria. Likewise, treatment of mice with PGZ for 12 weeks at a dose that has demonstrated to have pharmacologic effects (10 mg PGZ/g/day) [14] resulted in a significant decrease in complex I, but not complex III, activity. These results are consistent with those obtained by others with PGZ and other TZDs [7,8,15] and with those we have previously reported in ob/ob mice [12]. Although some authors found that this effect of PGZ on complex I activity was not dose-dependent [15], we found a clear relationship between the dose of PGZ and the decrease in complex activity (Figure 1A). In contrast, we found no effects of PGZ on complex II, IV, or V activity.

Mechanisms by which PGZ decreases complex I and III activity are not well understood. Complex I transfers two electrons from NADH to ubiquinone, represents the main entrance site for electrons into the MRC, and translocates four protons across the mitochondrial inner membrane [16]. It is composed of two arms forming an L-shaped structure. One arm is hydrophobic and is embedded in the inner mitochondrial membrane, whereas the other arm is hydrophilic and protrudes into the mitochondrial matrix. While the hydrophilic arm is formed by subunits involved in electron transfer from NADH to ubiquinone, the membrane arm is responsible for the proton translocation [17].

Activity of complex I can be reduced as a result of a large number of factors including mutations in complex I subunits [18], physiological molecules, such as nitric oxide [19], drugs, such as rotenone and TZD [7,15,20], or oxygen or nitrogen derived reactive substances such as peroxynitrite [21]. Moreover, defects in the assembly/stability of this complex may also result in a reduced activity of complex I.

**Figure 6:**
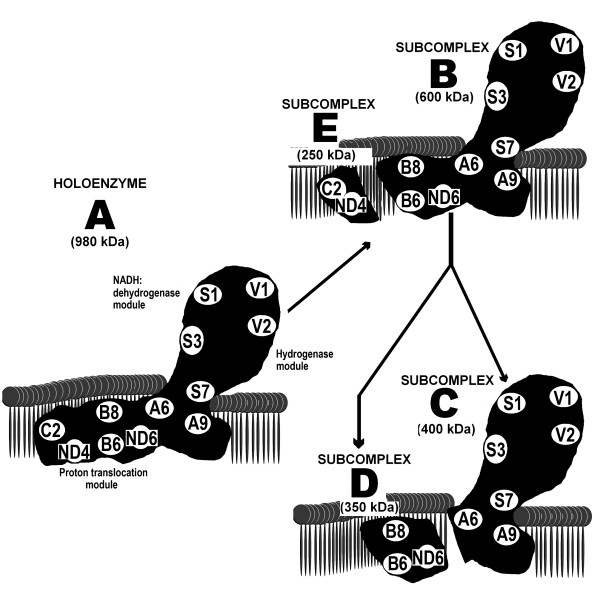
**Schematic representation of the pioglitazone (PGZ)-induced fragmentation of complex I (A) into four subcomplexes.** In the presence of PGZ, the membranous arm of complex I releases a fragment of 250 kDa **(E)**. As a result of this release, one subcomplex of about 600 kDa **(B)** is formed. Subsequently, the membrane arm of subcomplex (B) releases a second fragment of 350 kDa **(D)**. Thereafter, the remaining complex I is limited to a subcomplex of 400 kDa **(C)**. Subcomplexes (B) and (C) contain the NADH:dehydrogenase module and consequently have in-gel dehydrogenase activity.

Our study shows that PGZ affects the stability of complex I as indicated by the presence of its subunits in the mature holoenzyme and four additional lower molecular weight subcomplexes with apparent molecular masses of 600, 400, 350, and 250 kDa, respectively. The assembly process of complex I is still not completely understood and a number of different assembly models have been proposed [9]. However, in our study, the presence of these four additional subcomplexes cannot be attributed to an effect of PGZ on the assembly process, in as much as we added the PGZ to mitochondria isolated from the liver of a normal mouse in which complexes are fully assembled as shown in control untreated proteins. Therefore, formation of these four subcomplexes may be the result of the fragmentation of complex I into subcomplexes. The subcomplex B of approximately 600 kDa of molecular weight has NADH:dehydrogenase activity (Figure 2B) and contains the same subunits as the mature holoenzyme A, except subunits NADUFC2 and MTND4 (Figure 3A,B), which appear in the small subcomplex E of about 250 kDa. Following the assembly model proposed by Mimaki *et al*. [9], these subunits are components of a subcomplex also containing subunit MTND5 that bind to the distal end of the membrane arm of complex I (Figure 6). Thus, subcomplexes B and E may be the result of the detachment of this distal end of the mature complex I. The subcomplex D, of about 350 kDa of molecular weight, contains subunits MTND6, NDUFB6 and NDUFB8 of the membrane arm of complex I, but not the remaining studied subunits. Therefore, this subcomplex may also correspond to a fragment that has been detached from the membrane arm of subcomplex B (Figure 6). The subcomplex C, of approximately 400 kDa of molecular weight and with NADH:dehydrogenase activity (Figure 2B), may be the remaining fragment of complex I after having lost subcomplexes E and D (Figure 6).

Mechanisms by which PGZ destabilizes complex I are unclear. A number of factors have been recognized to be implicated in the assembly/stabilization of complex I, including complex III [22], NDUFAF1 (CIA30) [23], NDUFAF2 (B17.2L) [24], NDUFA9 complex I subunit [25], FOXRED1 [26], and prohibitin [27], among others. Although our study shows that *in vitro* treatment of isolated mitochondria with PGZ markedly altered these proteins, we do not believe that the PGZ-induced disassembly of complex I resulted from the effects of PGZ on these stabilizing proteins, as some of these proteins (NDUFAF1, NDUFAF2) dissociate before complex I is completely assembled and are not found in the mature holoenzyme [23,24]. Prohibitin, a protein that likely prevents complex degradation by AAA-metalloproteinases [27], decreased in parallel with fully assembled complex I (Figure 4A). However, our study cannot differentiate whether PGZ-induced complex I fragmentation was the result of the reduced amount of prohibitin or, on the contrary, prohibitin bound to complex I was reduced because PGZ had decreased fully assembled complex I. In the case of NDUFAF1, a chaperone not found associated with mature complex I, PGZ induced its binding to a new complex of 600 kDa, likely the subcomplex B formed after fragmentation of complex I. (Figures 4A,B, and 6). The effect of PGZ on FOXRED1 is difficult to interpret as very little is known about the function of this protein [26]. We are not aware of other studies that have been published on the effects of TZDs on prohibitin or other chaperone proteins.

Our study also shows the binding of radiolabeled PGZ to mature complex I and subcomplexes of 600 and 400 kDa of molecular weight (Figure 4C). Little information is available in the literature concerning the interaction of TZD with complex I proteins, although it has been suggested that PPARγ ligands interact directly with this complex [7]. Only Colca *et al*. have studied the interaction of PGZ with mitochondrial membranes and found that radiolabeled PGZ binds to a 17-kDa mitochondrial protein [28]. Our study shows the binding of PGZ to complex I components, particularly to NDUFA9, NDUFB6, and NDUFA6 subunits (Figure 4). These subunits are hydrophobic and are situated in the inner mitochondrial membrane. TZD, like other inhibitors of this complex [29], are highly lipophilic and bind mitochondrial membrane [30]. Thus, we could speculate that molecular changes induced by the binding of PGZ to these subunits may destabilize complex I resulting in the progressive fragmentation of its membrane arm.

The decrease in complex III activity observed after *in vitro* treatment of mitochondria with PGZ may be ascribed to the fact that complexes I and III interact physically to form supercomplexes that improves electron transfer between them [31] and stabilizes complex III [18]. Therefore, PGZ-induced disassembly of complex I may also indirectly compromise complex III stability and activity. In accordance with these facts, our study shows that *in vitro* addition of PGZ to isolated mitochondria reduced not only the amount of complex I, but also the formation of supercomplexes CI+CIII_2_+CIV_n_ and CI+CIII_2_ (Figure 2E). In contrast, supercomplexes CI+CIII_2_+CIV_n_ and CI+CIII_2_ were clearly increased in mice treated with PGZ for 12 weeks. This increase in the formation of supercomplexes in PGZ-treated mice, may justify why complex III activity remained normal in these animals (Figure 2F). This elevated accumulation of complex I and III to form supercomplexes might be a consequence of the association of MRC complexes to form more efficient respiratory structures [32] that might compensate the reduced activity of complex I and III of the MRC. This increase in supercomplex formation may also contribute to the elevated synthesis of complex I and III subunits.

The present study also shows that treatment of mice with PGZ for 12 weeks increased the amount of fully assembled complex I (Figure 2C), although its activity was significantly decreased (Figures 1B and 2D). Similar to what occurred in *in vitro* studies, these *in vivo* studies demonstrated the presence of two additional subcomplexes of 600 kDa and 400 kDa (Figure 2A) indicating that complex I was also disassembled. The increased amount of fully assembled complex I found in PGZ-treated mice may be ascribed to the fact that gene expression of nuclear DNA-encoded complex I subunits was significantly increased (Figure 5A). Our study shows that PGZ increased gene expression of complex I and, to a lesser extent, complex III subunits in mice and HepG2 cells (Figure 5A,B). These effects may be interpreted as a compensatory response to the decreased activity of these complexes found after treatment with PGZ. Transcription of MRC subunits is coordinated with energetic needs of the cells. Thus, the cellular pool of ATP, the primary end product of oxidative phosphorylation, plays a key role in the control of nuclear and mitochondrial transcription [33,34]. In the present study we clearly show that incubation of HepG2 cells with PGZ significantly decreased cellular content of ATP (Figure 4C). Therefore, it might be expected that decreased ATP formation as a result of the PGZ-induced complex I inhibition might lead to an increased transcription of some components of the MRC. The lack of increased expression of mtDNA-encoded subunits in PGZ-treated mice may be attributed to the reduced ability of cells to repair mtDNA damage [35]. A large number of transcription factors have been shown to be involved in the regulation of genes encoding MRC proteins and assembly factors, including *stimulatory protein 1* (*Sp1*), *ERRα*, and *transcriptional coactivator PPARγ* (*PGC1α*), among others [36]. In particular, transcriptional coactivator PGC1α plays a critical role in adaptation mechanisms to caloric restriction and ATP reduction [37]. In our study, we show that treatment of mice with PGZ or exposition of HepG2 cells to PGZ induced the expression of transcription factors *Sp1* and particularly *PGC1α* (Figure 5D,E). Very little information is available about the effects of TZD on gene expression of components of the MRC and its regulatory factors. Strum *et al*. found that rosiglitazone increases expression of genes regulating mitochondrial biogenesis in mice and that this effect was associated with an increased level of ERRα mRNA but not of PGC1α mRNA [38]. However, Boden *et al*. found that troglitazone and other TZDs, upregulated gene expression and synthesis of proteins involved in electron transport and oxidative phosphorylation in subcutaneous fat [39], and Mensink *et al*. demonstrated that rosiglitazone increased *PGC1α* gene expression in patients with type 2 diabetes mellitus [40].

## Conclusions

In summary, in the present study we show that PGZ binds complex I subunits, likely NDUFA9, NDUB6, and NDUFA6, which may induce disassembly of this complex, reduces its activity, depletes cellular ATP, and, in mice and HepG2 cells, upregulates nuclear DNA-encoded gene expression of complex I and III subunits. Elucidation of the molecular mechanisms by which PGZ binding provokes disassembly of complex I requires further studies.

## Methods

### *In vitro* experiments

The effects of PGZ on the MRC were explored in mitochondrial complexes isolated from the liver of C57BL/6J mouse and from the hepatocellular carcinoma cell line (HepG2; American Type Culture Collection (ATCC)) cultured in Dulbecco’s modified Eagle medium (Lonza Iberica SA, Barcelona, Spain) with 10% fetal calf serum, 1% l-glutamine, 1% penicillin, 1% streptomycin, 1% fungizone.

### *In vivo* experiments

All procedures on mice were carried out in accordance with the Spanish Guidelines for the Care and Use of Laboratory Animals. The 6-week-old male C57BL/6J mice were purchased from Charles River Laboratory (Charles River Laboratories España SA, Santa Perpetua de la Mogoda, Spain). Animals were housed at constant room temperature (23°C) (n = 3 per cage) under 12 h light/dark cycles with *ad libitum* access to water and standard laboratory mouse chow. A total of 20 mice were distributed into two groups: group I (control) included 10 untreated mice; group II (PGZ) consisted of 10 mice treated with 10 mg/kg/day of PGZ by daily gavage (Hartmann Analytic, Grupo Taper SA, Alcobendas, Spain) for 12 weeks. Food but not water was withdrawn overnight before mice were killed. The last PGZ dose was administered 20 h before tissue harvesting. Following the treatment, animals were anesthetized and killed at 18 weeks of age, and the liver was rapidly harvested for further analysis.

### Extraction of MRC complexes from mouse liver

Mitochondrial isolation from mouse liver samples was carried as described by Schägger *et al*. [41] with some modifications. Briefly, liver samples (50 mg; wet weight) were homogenized in a tightly fitting glass-teflon homogenizer with about 500 μl of homogenizing buffer, buffer A (440 mM sucrose, 20 mM 3-(*N*-morpholino)propanesulfonic acid (MOPS), 1 mM ethylenediaminetetra-acetic acid (EDTA), pH 7.2, and 0.2 mM phenylmethylsufonyl fluoride). The sediment was homogenized in 500 μl of buffer B (500 mM NaCl, 10 mM MOPS, pH 7.2). After centrifugation at 20,000 *g*, 150 μl of the 1 M aminocaproic acid, 50 mM Bis-Tris–HCl buffer C (pH 7.0) and 20 μl of Brij 35 detergent solution was added. Mitochondrial pellets from mouse liver were suspended in an appropriate volume of buffer D (1 M 6-amiohexanoic acid, 50 mM Bis-Tris/HCl, pH 7.0), and the membrane proteins were solubilized by the addition of the indicated detergent and incubated for 10 minutes in ice. After centrifugation for 15 minutes at 100,000 rpm at 4°C, the supernatant was collected, and one-third of the final volume of the sample of 5% Serva Blue G dye in 1 M 6-amiohexanoic acid was added prior to loading. The following detergents were used (a) 3% (w/v) *n*-dodecyl β-d-maltoside (DDM) for preparation of native MRC complexes and (b) 4 g/g protein digitonin (DIG) to detect the supercomplexes.

### MRC activity assays

Frozen liver tissues (50 to 70 mg) were homogenized with 15 vol. of 20 mmol/L KP buffer (K_2_HPO_4_), pH 7.4, and centrifuged at 800 g for 10 minutes. MRC enzymes and citrate synthase (CS) activities were measured in a DU-650 spectrophotometer (Beckman Instruments, Palo Alto, CA, USA). Incubation temperatures were 30°C for complexes I, II, III, V and CS, and 38°C for complex IV. Enzyme activities were performed in supernatants as described elsewhere [42], expressed as nM of substrate used per minute per mg protein and, to correct for the hepatic content of mitochondria, referred as a percentage of the specific activity of CS. Enzyme assays were performed in quintuplicate.

### Assessment of full assembly of MRC complexes

MRC complexes from were isolated by one-dimensional BN-PAGE as described elsewhere [21]. Following electrophoresis, proteins were transferred to a polyvinyl difluoride membrane (0.45-μm pore size) (Immobilon-P transfer membrane; Millipore Co., Bedford, MA, USA). Western blotting of these proteins was performed using primary antibodies against complex I subunits NDUFA6, NDUFA9, NDUFC2, NDUFV1, NDUFV2, NDUFS1, NDUFS7, NDUFS3, NDUFB6, NDUFB8, MTND6, complex II subunit 70 (SDHA), complex III subunit core 2 (UQCRC2), complex IV subunit COX-IV (COX4) and complex V subunit α (ATP5A1) (Molecular Probes Inc., Eugene, OR, USA) on blocking buffer for 2 h. After washing, blots were incubated for 1 h with peroxidase-conjugated antibody as a secondary antibody, prepared at a 1:5,000 dilution (Molecular Probes Inc.). Immunoreactive material was visualized by chemiluminescence (ECL, Western Blotting Detection; GE Healthcare, Madrid, Spain) according to the manufacturer’s instructions. The blot was finally exposed to Hyperfilm MP (Amersham, GE Healthcare).

### Second-dimension electrophoresis for assessing complex subunits

For second-dimension SDS-PAGE, we followed the procedure previously described [21]. Following electrophoresis, proteins were transferred to a polyvinyl difluoride membrane. Western blotting was performed using primary antibodies against complex I subunits NDUFA9, NDUFS3, NDUFB6 (Molecular Probes Inc.), NDUFV1, NDUFV2, NDUFS1, NDUFA6, MTND1, MTND2, MTND4, and MTND4L (Santa Cruz Biotechnology, Inc. Santa Cruz, CA, USA), complex II subunit SDHA, complex IV subunit COX4, complex V subunit ATP5A1 (Molecular Probes Inc.) in blocking buffer for 2 h. After washing, blots were treated as indicated above.

### In-gel activity assays

MRC complexes were separated by one-dimensional BN-PAGE as described above and complex I in-gel activity was measured as described by Nijtmans *et al*. [43].

### Quantitative real-time polymerase chain reaction

Total RNA was extracted from cultured HepG2 cells and from the liver of control and PGZ-treated C57BL/6J mice using the TRI-Reagent (Sigma-Aldrich, Steinheim, Germany) according to the manufacturer’s instructions. RNA was treated with DNase I to remove DNA contamination (Sigma-Aldrich). cDNA was generated from 1 μg sample RNA using First Strand cDNA Synthesis Kit for RT-PCR (Roche Applied Science, Indianapolis, IN, USA) at 25°C, 5 minutes; 42°C, 60 minutes; 95°C, 5 minutes, and 4°C, 5 minutes. Quantitative real-time PCR was performed on a Light Cycler 1.0 (Roche Applied Science) in 20 μl with 50 ng cDNA, 0.5 μM primers, and 2 μl FastStart DNA Master SYBR Green I (Roche Applied Science). Data from the real-time, quantitative PCR were analyzed following the method described elsewhere [21]. Sequences of primers used in these experiments are shown in Table 1. Expression of complex I subunits was normalized to that corresponding β-actin. The amplification conditions were 45 cycles of denaturation at 95°C for 10 s, annealing at 57°C for 5 s, and extension at 72°C for 20 s. The correct size and purity of the amplified products was verified by agarose gel electrophoresis.

**Table 1:** Primers used in quantitative real-time polymerase chain reaction

**Primer name**	**Direction**	**Sequence**
Mouse NDUFA9 (39 kDa)	Sense	5′-CAT TAC TGC AGA GCC ACT-3′
	Antisense	5′-ATC AGA CGA AGG TGC ATG AT-3′
Mouse NDUFB6 (17 kDa)	Sense	5′-ATA ACT TTT TGC GGG ACG GG-3′
	Antisense	5′-CAG GAA AAT CTC TCA TTG GTG-3′
Mouse NDUFS3 (30 kDa)	Sense	5′-AGG AAC ATG GCG GCG GCT GC-3′
	Antisense	5′-ATT TCA GCC ACA TAC TCT CC-3′
Mouse MTND1 (ND1)	Sense	5′-TGC ACC TAC CCT ATC ACT C-3′
	Antisense	5′-ATT GTT TGG GCT ACG GCT C-3′
Mouse MTND2 (ND2)	Sense	5′-ATG AGT AGG CCT GGA ATT C-3′
	Antisense	5′-ATC AGA AGT GGA ATG GGG C-3′
Mouse MTND4 (ND4)	Sense	5′-ATA ATT ATA ACT AGC TCA ATC TGC-3′
	Antisense	5′-TCG TAG TTG GAG TTT GCT AG-3′
Mouse MTND4L (ND4L)	Sense	5′-CTC ACC ATA GCC TTC TCA C-3′
	Antisense	5′-CGT AAT CTG TTC CGT ACG TG-3′
Mouse β-actin	Sense	5′-ATG GAT GAC GAT ATC GCT G-3′
	Antisense	5′-GTT GGT AAC AAT GCC ATG TTC-3′
Mouse UQCRC1 (core 1)	Sense	5′-CCT ACA GCA CTC GAG AGC AC-3′
	Antisense	5′-AGG TGT GCC CTG GAA TGC TG-3′
Mouse UQCRC2 (core 2)	Sense	5′-TCC CTC AAA GTT GCC CC-3′
	Antisense	5′-GCA AGA CGT AGT AAA TGT GAG-3′
Mouse UQCRFS1 (FeS)	Sense	5′-GAT GTC AAG GTG CCC GAC TT-3′
	Antisense	5′-GAT CTC GAT CTT CGA CAT GG-3′
Mouse SDHA (70 kDa)	Sense	5′-CAT ACT GTT GCA GCA GCA CAG G-3′
	Antisense	5′-CCA CCA AAT GCA CGC TGA TA-3′
Mouse COX4 (COX IV)	Sense	5′-GAG CAC ATG GGA GTG TTG TG-3′
	Antisense	5′-CTG TCT TCC ATT CAT TGG TGC C-3′
Mouse PGC1α	Sense	5′-GAG ACT TTG GAG GCC AGC AAG-3′
	Antisense	5′-CCA AGG GTA GCT CAG TTT ATC AG-3′
Mouse ERRα	Sense	5′-GAC AGT CCA AAG GGT TCC TCA G-3′
	Antisense	5′-CTG GAT GGT CCT CTT GAA GAA GGC-3′
Mouse SP1	Sense	5′-CTG CCA GCT TGG TGT CAT CAC-3′
	Antisense	5′-CTG ACT TCC TTG CAG CGA GC-3′
Human NDUFA9 (39 kDa)	Sense	5′- GAT TGT GGC CAC TGT GTT TGG-3′
	Antisense	5′-CTC CAG CTT CCT TGG ACA GT-3′
Human NDUFS3 (30 kDa)	Sense	5′-GTC AGA CCA CGG AAT GAT GTG-3′
	Antisense	5′-CTC AAA ACG GTT TTG CCG AG-3′
Human NDUFB6 (17 kDa)	Sense	5′-CTG CAG CAG CTG CGA GA-3′
	Antisense	5′-GAA TAA TCC AGA CAG GTA CAA G-3′
Human UQCRC1 (core I)	Sense	5′-GTT AGC CTG CTG GAC AAC G-3′
	Antisense	5′-CTT GAT GTA GTA AGC TGT GTG C-3′
Human UQCRC2 (core II)	Sense	5′-CCA AAT GGC TTG GTG ATT GC-3′
	Antisense	5′-CAT TAG AAT ATC AAC ATC ACC CC-3′
Human UQCRFS1 (FeS)	Sense	5′-GCC TGT GTT GGA CCT GAA G-3′
	Antisense	5′-CTG GAA ACG AAC TGG GTG AC-3′
Human COX4 (COX IV)	Sense	5′-CAG GGT ATT TAG CCT AGT TGG-3′
	Antisense	5′-CTC CTT GAA CTT AAT GCG ATA C-3′
Human SDHA (70 kDa)	Sense	5′-GGC TTG CGA GCT GCA TTT GG-3′
	Antisense	5′-GTT CTG CTA AAC GGC ATG CCA-3′
Human PGC1α	Sense	5′-GCA AAC TTG CTA GCA GTC CTC-3′
	Antisense	5′-GGT ACT GAG CCA ACT GCA TTC-3′
Human ERRα	Sense	5′-GAG TGT GAG ATC ACC AAG CG-3′
	Antisense	5′-CAC ATG AGA CAC CAG TGC ATT C-3′
Human SP1	Sense	5′-CAG CAG AAT TGA GTC ACC CAA T-3′
	Antisense	5′-CAT ACT GCC CAC CAG AGA C-3′

### Western blot analysis

MRC complexes were isolated by BN/SDS-PAGE as described above and the filters containing proteins were incubated with appropriated polyclonal antibodies against prohibitin, FOXRED1, and NDUFAF1 (Santa Cruz Biotechnology, Inc. Santa Cruz, CA, USA). Signals were detected using the ECL Western Blotting Detection Reagent (Amersham Ibérica, Madrid, Spain).

### Measurement of total intracellular ATP content

HepG2 cells were grown to confluence in 75 cm^2^ tissue culture flasks (approximately 1.0×10^6^ cells) and detached using trypsin. After washing in phosphate-buffered saline (PBS), cell pellets were divided into two portions for duplicate experiments. Before ATP determination, pellets were homogenized using perchloric acid and centrifuged ice cold at 15,000 *g* for 2 minutes to pellet insoluble materials. Supernatant was collected and 30 μl was added to a 96-well plate, then brought up to 50 μl with ATP assay buffer. ATP reaction mix and ATP measurement was performed using the ATP Colorimetric/Fluorometric Assay Kit (BioVision Research Products, Linda Vista, CA, USA) according to the manufacturer’s protocol.

### Binding of [^3^H]PGZ to complex I subunits

A total of 100 μg of liver mitochondria isolated from a C57BL/6J mouse were incubated with 10 μM [^3^H]PGZ (PGZ ^3^H(G) hydrochloride; Hartmann Analytic, Grupo Taper SA, Alcobendas, Spain) at 27°C for 30 minutes. Afterwards, proteins were separated in parallel, simultaneously, on three one-dimensional BN-PAGE as described above. Following electrophoresis, proteins from one of these three gels were transferred to a polyvinyl difluoride membrane (0.45-μm pore size) (Immobilon-P transfer membrane; Millipore) as previously described [20]. Western blotting of these proteins was performed using primary antibodies against complex I subunit 39 (NDUFA9), on blocking buffer for 2 h (Molecular Probes Inc.). The second gel was used to visualize radioactive subunits by autoradiography. The third gel was utilized to visualize complex I in-gel activity. Likewise, subunits of complex I were separated in parallel, simultaneously, on two SDS/BN-PAGE gels as described above. Following second-dimension electrophoresis, subunits from one of these two gels were transferred to a polyvinyl difluoride membrane as previously described and identified using antibodies against subunits NDUFA6, NDUFA9, NDUFB6, NDUFS1, NDUFV1, NDUFV2, and NDUFA2. The second gel was used to visualize radioactive subunits by autoradiography.

### Coimmunoprecipitation analysis

Liver mitochondria were isolated from liver homogenates by differential centrifugation as described elsewhere [13]. Mitochondrial pellets were treated with 10 μM [^3^H]PGZ for 30 minutes and lysed in radioimmunoprecipitation assay (RIPA) buffer (20 mM Tris/HCl, pH 7.5; 150 mM NaCl; 2 mM EDTA; 1% sodium deoxycholate; 1% Triton X-100; 0.25% SDS). A total of 800 μg of mitochondrial lysates were incubated with 2 μg monoclonal antibody (NDUFA9; NDUFV2; NDUFS1; NDUFA6; NDUFB6; COX1; core 1) at 4°C overnight. Following incubation, the immune complexes were precipitated with 50 μl protein G (NDUFA9, NDUFB6, core 1, COX1) or protein A (NDUFA6, NDUFS1, NDUFV1) MicroBeads (Miltenyl Biotec Inc. Auburn, CA, USA) for 4 h on ice. Columns were placed in the magnetic field of the μMACS separator and rinsed with 200 μl PBS buffer (pH 7.5). Precipitates were placed onto the columns and let to run through. The columns were rinsed four times with 200 μl PBS buffer and once with 100 μl low salt buffer (20 mM Tris/HCl (pH 7.5)). Afterwards, 20 μl of preheated 95°C hot 1 × SDS gel loading buffer (50 mM Tris/HCl (pH 6.8); 50 mM dithiothreitol; 1% SDS; 0.005% bromophenol blue; 10% glycerol) was applied to the column and incubated for 5 minutes at room temperature. Following incubation, 50 μl of preheated 95°C hot SDS gel loading buffer was added and elute was collected as immunoprecipitate. This was separated into two aliquots. In one of these, the radioactivity of the sample was quantitated by scanning laser densitometry (Desk TopTM Scanner Plus, Amersham Pharmacia Biotech). The second aliquot was electrophoresed in a 10% acrylamide gel and transferred to a nitrocellulose membrane. Subunits were analyzed by western blot. In addition, the individual bands in the blot containing subunits were excised and their radioactivity was measured by liquid scintillation spectrophotometry.

### Statistical analysis

Statistical analysis was performed using SPSS Statistical Software for Windows, version 9 (SPSS Inc. Chicago, IL, USA). The unpaired t test was used to assess the significance of differences between means. Each *in vitro* experimental condition was repeated three to four times. Quantitative data are expressed as the mean ± SD unless otherwise mentioned. *P* values <0.05 were considered significant.

## Abbreviations

ATP5A1: Subunit α complex V; BN-PAGE: Blue native polyacrylamide gel electrophoresis; CS: Citrate synthase; ERRα: Estrogen related receptor α; FADH2: Reduced flavin adenine dinucleotide; MRC: Mitochondrial respiratory chain; mtDNA: Mitochondrial DNA; nDNA: Genomic DNA; PGC-1α: PPARγ coactivator 1α; PGZ: Pioglitazone; PPARγ: Peroxisome proliferator-activated receptor γ; SDHA: Subunit 70 kDa complex II; Sp1: Specific protein-1; TZD: Thiazolidinediones; UQCRC2: Subunit core 2 complex III.

## Competing interests

The authors declare that they have no competing interests.

## Authors’ contributions

IG-R performed many of the experiments and contributed to design, analysis and interpretation of data. PS-M was involved in the acquisition, analysis and interpretation of data, critical revision of the manuscript for important intellectual content. DF-M performed many of the experiments and analyzed and interpreted data. He also reviewed and approved the final version of the manuscript. TM-Y contributed by designing the study, in acquisition of data, and critical revision of the manuscript. JAS-H was involved in the conception, design and supervision of the study, in the analysis and interpretation of data, and writing the manuscript. All authors read and approved the final manuscript.
